# pH-Responsive Nanophotosensitizer Boosting Antibacterial Photodynamic Therapy by Hydroxyl Radical Generation

**DOI:** 10.3390/nano15141075

**Published:** 2025-07-10

**Authors:** Peilin Tian, Xianyue Bai, Jing Feng, Luyao Xu, Shihao Xu, Xiaoya Yu, Caiju Fan, Qian Su, Jiaxing Song, Cuixia Lu

**Affiliations:** 1 Guangxi Key Laboratory of Special Biomedicine, School of Medicine, Guangxi University, Nanning 530004, China; tianpl1216@st.gxu.edu.cn (P.T.); bxy@st.gxu.edu.cn (X.B.); fengj@st.gxu.edu.cn (J.F.); luluna@st.gxu.edu.cn (L.X.); xsh@st.gxu.edu.cn (S.X.); 2024203060025@whu.edu.cn (X.Y.); fcj@hnu.edu.cn (C.F.); 2237030113@st.gxu.edu.cn (Q.S.); 2 Cell and Immunology Laboratory, Medical Research Centre, School of Life Sciences and Medical Engineering, Guangxi Medical University, Nanning 530021, China

**Keywords:** antimicrobial photodynamic therapy, pH-responsive nanomaterial, antibiotic resistance, Fenton reaction

## Abstract

In this study, a pH-responsive nanophotosensitizer (MT@Ce6) was rationally developed by strategic integration of MIL-101 (Fe)-NH_2_ metal–organic framework with tannic acid (TA) and chlorin e6. This nanocomposite exhibits pH-responsive degradation in acidic microenvironments, facilitating Fe^3+^ release and subsequent reduction to Fe^2+^ that catalyzes Fenton reaction-mediated hydroxyl radical (•OH) generation. This cascade reaction shifts reactive oxygen species (ROS) predominance from transient singlet oxygen (^1^O_2_) to the long-range penetrative •OH, achieving robust biofilm disruption and over 90% eradication of methicillin-resistant *Staphylococcus aureus* (MRSA) under 660 nm irradiation. In vivo evaluations revealed accelerated wound healing with 95% wound closure within 7 days, while species-selective antibacterial studies demonstrated a 2.3-fold enhanced potency against Gram-positive bacteria due to their unique peptidoglycan-rich cell wall architecture. These findings collectively establish a microenvironment-adaptive nanoplatform for precision antimicrobial interventions, providing a translational strategy to address drug-resistant infections.

## 1. Introduction

The emergence of antibiotic-resistant pathogens such as MRSA has underscored the urgent need to develop novel antibacterial strategies. The World Health Organization categorizes MRSA as a “high-priority antimicrobial-resistant pathogen” with hospital-acquired infections exhibiting mortality rates as high as 20–30% [1]. However, the traditional antibiotic development pipeline has nearly stagnated, with only two new classes of antibiotics introduced over the past three decades [2], necessitating disruptive technologies to transcend current antibacterial paradigms.

Following Tappeiner’s proposal of the term “photodynamic therapy” to describe light-dependent treatments [3,4], antimicrobial photodynamic therapy (PDT) has demonstrated significant potential in managing oral infections and chronic wounds [4,5,6]. Antimicrobial PDT employs photosensitizers to generate ROS under light irradiation, inducing microbial death through ROS-mediated oxidation of lipids, proteins, and nucleic acids within microorganisms [5,7,8]. Its broad-spectrum antimicrobial efficacy and low resistance induction risk have positioned PDT as a promising therapeutic approach [9,10]. However, conventional photosensitizers such as methylene blue and porphyrin derivatives suffer from several critical drawbacks. First, they predominantly generate short-lived ^1^O_2_, which has limited diffusion distance and cannot penetrate dense biofilm matrices, thereby reducing antibacterial efficacy in complex infection environments [11,12,13]. Second, their excessive oxygen dependency markedly lowers therapeutic performance under hypoxic conditions [14,15,16]. Third, non-specific oxidative damage often induces host cytotoxicity and restricts the therapeutic window. Iron-based metal–organic frameworks (MOFs), particularly MIL-101 (Fe) and its amino-functionalized derivative MIL-101 (Fe)-NH_2_, have emerged as promising platforms for biomedical applications [17,18,19]. The MIL-101 (Fe)-NH_2_ metal–organic framework is constructed from trimeric iron (III) octahedral clusters bridged by 2-aminoterephthalate linkers, yielding a three-dimensional porous network with exceptionally large cages (∼29–34 Å diameter) [20]. The presence of pendant –NH_2_ groups offers multiple benefits over non-functionalized MOFs: it enhances framework hydrophilicity, enables facile post-synthetic modification (e.g., Schiff-base conjugation), and provides coordination sites for guest molecules [21]. These features make MIL-101 (Fe)-NH_2_ particularly attractive for loading and controlled release of therapeutic cargos as well as for catalytic applications where surface functionality is crucial. Moreover, the Fe-O clusters within its framework exhibit intrinsic Fenton-like catalytic activity, efficiently converting H_2_O_2_ into •OH under mildly acidic conditions. These characteristics have been widely exploited in chemodynamic therapy, drug delivery, and biosensing; however, their integration into PDT platforms remains at an early stage of investigation. Chlorin e6 (Ce6) is a naturally derived chlorophyll metabolite featuring a reduced porphyrin macrocycle with strong absorption in the red region (λ_max_ ≈ 660 nm) and high singlet-oxygen quantum yield [22]. Its carboxylate and hydroxyl substituents facilitate coordination to metal centers or hydrogen bonding with functional groups (e.g., –NH_2_) on the MOF surface. As a well-established photosensitizer in PDT, Ce6 exhibits excellent tissue penetration and minimal dark toxicity, making it an ideal candidate for incorporation into MOF-based nanoplatforms.

TA, a naturally occurring polyphenol, has garnered significant attention due to its multifunctional coating and reducing capabilities [23,24]. When applied to MOF surfaces, TA serves as a protective layer that prevents premature framework degradation, enhances colloidal stability, and introduces stimuli-responsive behavior [25]. Notably, under acidic conditions, TA can rapidly reduce Fe^3+^ to Fe^2+^, thereby amplifying in situ Fenton-type radical generation. Therefore, TA-containing MOF rapidly reduces Fe^3+^ to ferrous ions Fe^2+^ under the mildly acidic conditions characteristic of infected wounds. Several studies have demonstrated that TA-coated iron oxide or MOF nanocomposites enhance •OH production in tumor chemodynamic therapy via acid-triggered Fe^2+^ release [26,27,28]. However, similar strategies have not yet been fully explored in the context of antimicrobial photodynamic therapy.

Herein, we present the rational design of a pH-responsive nanophotosensitizer (MT@Ce6) through the strategic integration of MIL-101 (Fe)-NH_2_, TA, and Ce6 into a unified platform. This construct establishes a PDT paradigm by simultaneously enabling microenvironment-triggered specificity and multimodal antimicrobial action. It is expected to greatly improve the efficacy to traditional PDT.

## 2. Materials and Methods

### 2.1. Materials and Reagents

2-NH_2_-H_2_BDC, FeCl_3_•6H_2_O, tannic acid, methylene blue (MB), and ethanol were purchased from Macklin Co., Ltd. (Shanghai, China). Ce6, N,N-dimethylformamide (DMF),FeSO_4_•7H_2_O, H_2_O_2_, and NaOH were obtained from Aladdin Co., Ltd. (Shanghai, China). 9,10-Anthracenediyl-bis(methylene)dimalonic acid (ABDA) was supplied by Yuanye Bio-Technology Co., Ltd. (Shanghai, China). 2′,7′-Dichlorodihydrofluorescein diacetate (DCFH-DA) was purchased from Solarbio Co., Ltd. (Beijing, China). Aminophenyl fluorescein (APF) was purchased from AAT Bioquest Inc. (Sunnyvale, CA, USA). BALB/c mice (6–8 weeks old) were obtained from Sippef BioTech Co., Ltd. (Beijing, China). MRSA, *Staphylococcus aureus*, and *E. coli* strains were purchased from the Guangdong Culture Collection Center (Guangzhou, China).

### 2.2. Synthesis of Nanocomposites

MIL-101 (Fe)-NH_2_ was prepared according to the procedure reported by Liang et al. [29,30]. 2-aminoterephthalic acid (2-NH_2_-H_2_BDC, 0.67 g) and FeCl_3_•6H_2_O (1.62 g) were employed to prepare MIL-101 (Fe)-NH_2_ via a solvothermal reaction. The two reagents were dissolved in a mixed solvent of DMF (25 mL) and deionized water (5 mL), ultrasonicated briefly, then transferred to a 50 mL Teflon-lined autoclave and heated at 110 °C for 24 h. After cooling to room temperature, the pale brown solid was centrifuged (8000 rpm, 10 min), washed three times with fresh DMF (20 mL each), soaked in ethanol (20 mL) at 60 °C for 12 h to exchange solvents, then collected by centrifugation and dried under vacuum at 60 °C for 12 h. For TA coordination, 50 mg of the dried MIL-101 (Fe)-NH_2_ powder was dispersed in deionized water (20 mL) with stirring, and a tannic acid solution (2 mg·mL^−1^ in water, 10 mL) was added dropwise over 10 min at pH ≈ 7 (adjusted with 0.1 M NaOH). The mixture was stirred at 25 °C for 6 h, yielding a brownish MOF/TA composite that was collected by centrifugation (8000 rpm, 10 min), washed twice with water (15 mL) and once with ethanol (15 mL), and dried under vacuum at 40 °C for 6 h. To load Ce6, Ce6 (1.0 mg·mL^−1^ in anhydrous ethanol, 10 mL) was added dropwise to a suspension of MOF/TA (20 mg in 10 mL anhydrous ethanol) under gentle stirring (400 rpm) at 25 °C in the dark. After 12 h, the MT@Ce6 nanocomposite was collected by centrifugation (8000 rpm, 10 min), washed three times with a 1:1 (*v*/*v*) mixture of anhydrous ethanol and deionized water (10 mL each), and finally dried under vacuum at 40 °C for 4 h to yield a dark green powder.

### 2.3. Material Characterization

Sample characterization methods were as follows: The morphology and size distribution of the nanocomposites were examined by transmission electron microscopy (TEM, JEOL JEM-2100, Hillsboro, OR, USA). Elemental mapping of C, N, and Fe was performed by dark-field scanning transmission electron microscopy (STEM) on a JEOL JEM-2100F (JEOL Ltd., Tokyo, Japan) equipped with an Oxford Instruments X-Max N 80 EDS detector (Oxford Instruments, Abingdon, UK). to confirm uniform element distribution. Fourier-transform infrared (FTIR) spectra were recorded using a Nicolet iS50 spectrometer (Thermo Fisher Scientific, Waltham, MA, USA) in the range of 400–4000 cm^−1^ to identify characteristic bond vibrations, such as Fe-O stretching near 579 cm^−1^ UV-Vis absorption spectra were obtained on a UV-2600 spectrophotometer (Shimadzu, Kyoto, Japan) to verify Ce6 incorporation. Prior to measurement, samples were dispersed in anhydrous ethanol (0.1 mg·mL^−1^), sonicated for 10 min, and analyzed using a 1 cm quartz cuvette. The appearance of Ce6-specific Soret and Q bands (≈400 nm and ≈660 nm, respectively) confirmed successful loading of Ce6 into the composite.

### 2.4. pH-Dependent Structural Evolution

MOF/TA nanocomposites (2 mg·mL^−1^) were incubated in 10 mM PBS at pH 5.0 or 7.4 (10 mL total volume) under gentle agitation (100 rpm) at 37 °C for 12 h. After incubation, 1 mL aliquots were collected, centrifuged (12,000 rpm, 10 min, 4 °C), and washed twice with deionized water. Pellets were resuspended in 100 µL deionized water, drop-cast onto copper TEM grids, air-dried, and imaged by TEM (JEOL JEM-2100) to evaluate pH-induced morphological changes.

### 2.5. Fe^3+^ Release Quantification

Following pH treatment (Section 2.3), MOF/TA suspensions were centrifuged (12,000 rpm, 10 min, 4 °C), and ~9 mL supernatant was filtered (0.22 µm). A 500 µL aliquot of filtrate was mixed with 400 µL acetate buffer (0.2 M, pH 4.5) and 50 µL 1,10-phenanthroline (1 mM in ethanol), then 50 µL hydroxylamine hydrochloride (0.5 M) was added to reduce Fe^3+^ to Fe^2+^. After vortexing and incubation for 30 min at ~25 °C, absorbance at 510 nm was measured. Fe^3+^ concentration was determined from a calibration curve (0–100 µM FeSO_4_•7H_2_O in acetate buffer, pH 4.5). All assays were performed in triplicate and expressed as µg Fe^3+^ per mg MOF/TA.

### 2.6. Hydroxyl Radical (•OH) Detection

In the MB decolorization assay, MOF/TA (100 µg·mL^−1^) was first suspended in 5 mL of 10 mM PBS (pH 7.4), and MB was added to a final concentration of 10 µg·mL^−1^ (from a 1 mg·mL^−1^ stock). Four reaction groups were prepared in triplicate—MB only (no MOF/TA, no H_2_O_2_), MB + MOF/TA (no H_2_O_2_), and MB + MOF/TA with 5, 10, or 15 mM H_2_O_2_—and all mixtures were incubated at 37 °C under static conditions. At 0 and 60 min, 200 µL aliquots were withdrawn from each tube, centrifuged at 12,000 rpm for 5 min at 4 °C, and 150 µL of the supernatant was transferred into a 96-well plate. The absorbance at 665 nm (A_665_) was measured on a Shimadzu UV-2600 spectrophotometer, and MB decolorization was calculated relative to the initial reading. Parallel controls lacking either MOF/TA or H_2_O_2_ confirmed that significant MB degradation only occurred when both catalyst and peroxide were present. *S. aureus* was cultured in LB at 37 °C with shaking (200 rpm) overnight. Cells were harvested by centrifugation and resuspended in PBS (pH 7.4) to an OD_600_ of 0.5 (~1 × 10^8^ CFU·mL^−1^). A 1 mL aliquot of this suspension was allocated to each treatment group (Ce6 equivalent = 2 µg·mL^−1^) and incubated in the dark at 37 °C for 30 min. After centrifugation (5000 rpm, 5 min) and removal of the supernatant, bacterial pellets were resuspended in PBS containing 5 µM APF (diluted from a 5 mM DMSO stock) and incubated in the dark at 37 °C for an additional 30 min. Cells were washed twice with PBS and transferred to glass-bottom confocal dishes. Samples were irradiated at room temperature (~25 °C) with a 660 nm laser (0.5 W·cm^−2^, sample distance ~3 cm) for 10 min. APF fluorescence was captured by confocal laser scanning microscopy (λ_ex_ = 488 nm; λ_em_ = 515–545 nm).

### 2.7. ^1^O_2_ Generation

ABDA (2 mM in DMSO) was diluted in 10 mM PBS (pH 7.4) to 50 µM. MOF/TA (100 µg·mL^−1^ in PBS, 1 mL total) was mixed with ABDA immediately before irradiation. Samples were irradiated with a 660 nm CW laser (0.5 W·cm^−2^ at the sample, distance ~3 cm) under magnetic stirring at ~25 °C. UV-Vis spectra (300–500 nm) were recorded at 0, 5, 10, 20, and 30 min post-irradiation. The decrease in A_359_ quantified ^1^O_2_ production. Control samples (ABDA + PBS, irradiated; MOF/TA + ABDA, dark) were included.

### 2.8. Intracellular ROS Detection

*S. aureus* was cultured in LB at 37 °C, 200 rpm overnight. Cells were pelleted (5000 rpm, 5 min), washed twice with 10 mM PBS (pH 7.4), and resuspended to OD_600_ = 0.5 (~1 × 10^8^ CFU·mL^−1^). A 1 mL aliquot was incubated with MT@Ce6 (Ce6 equivalent = 2 µg·mL^−1^) in the dark at 37 °C for 30 min. After pelleting (5000 rpm, 5 min), bacteria were resuspended in 1 mL PBS containing DCFH-DA (10 µM; from 1 mM DMSO stock) and incubated in the dark at 37 °C for 30 min. Cells were washed twice with PBS, transferred to glass-bottom confocal dishes, and irradiated with a 660 nm laser (0.5 W·cm^−2^ at the sample, ~3 cm) for 10 min at ~25 °C. Intracellular fluorescence (DCF; λ_ex_ = 488 nm, λ_em_ = 525 ± 25 nm) was captured by confocal laser scanning microscopy. Fluorescence intensity was quantified in three random fields per sample using ImageJ (version 1.53c, Win64; National Institutes of Health, Bethesda, MD, USA).

### 2.9. Membrane Damage Analysis

Bacterial suspensions treated with MT@Ce6 (Ce6 equivalent = 2 µg·mL^−1^) were irradiated with a 660 nm laser (0.2 W·cm^−2^ at the sample, ~3 cm) for 10 min. After irradiation, cells were collected (5000 rpm, 5 min) and fixed in 2.5% glutaraldehyde in 10 mM PBS (pH 7.4) at 4 °C for 2 h. Fixed cells were washed three times with PBS, dehydrated through graded ethanol (30, 50, 70, 90, 100% *v*/*v*; 10 min each), air-dried, sputter-coated with gold, and then imaged by field-emission scanning electron microscopy on a Carl Zeiss Sigma 500 SEM (Carl Zeiss AG, Oberkochen, Germany). to assess membrane integrity. Untreated and light-only controls were included.

### 2.10. Antibacterial Efficacy

Following photodynamic treatment (MT@Ce6 at Ce6 equivalents of 0, 1, 2, or 2 µg·mL^−1^ plus 660 nm irradiation at 0.2 W·cm^−2^ for 10 min), bacterial suspensions were serially diluted in sterile PBS (10^−1^ to 10^−5^). Aliquots of 100 µL from each dilution were spread onto LB agar plates in triplicate and incubated at 37 °C for 24 h.

### 2.11. Animal Model and Wound Infection

All animal procedures were approved by the Guangxi University Animal Care and Use Committee. BALB/c mice (6–8 weeks old, 18–22 g) were housed under standard conditions (12 h light/dark cycle, 22 ± 2 °C, ad libitum access to food and water). Animals were anesthetized via inhalation of 2% isoflurane, and dorsal hair was removed. A full-thickness excisional wound (6 mm diameter) was created on each mouse using a sterile biopsy punch. Immediately afterward, 20 µL of a *S. aureus* suspension (10^6^ CFU in PBS) was applied directly onto the wound bed.

### 2.12. Treatment Protocol and Evaluation

Mice were subjected to one of three protocols: no treatment (untreated control), light only (660 nm irradiation at 0.5 W·cm^−2^ for 10 min), or MT@Ce6 plus light. For the MT@Ce6 + light group, MT@Ce6 (Ce6 equivalent, 2 µg·mL^−1^ in PBS; 50 µL per wound) was applied topically, then, after a 30 min absorption period, the wound area was irradiated with a 660 nm laser (0.5 W·cm^−2^ at the sample surface; distance ≈ 3 cm) for 20 min. During irradiation, non-wounded skin was shielded to restrict exposure to the wound bed. Wound area photographs were acquired on days 0, 3, 5, and 7 using a digital camera with a metric ruler placed adjacent to the wound. Wound boundaries were traced manually in ImageJ to calculate area (mm^2^).

## 3. Results

### 3.1. Synthesis and Characterization of Nanocomposites

**Figure 1:**
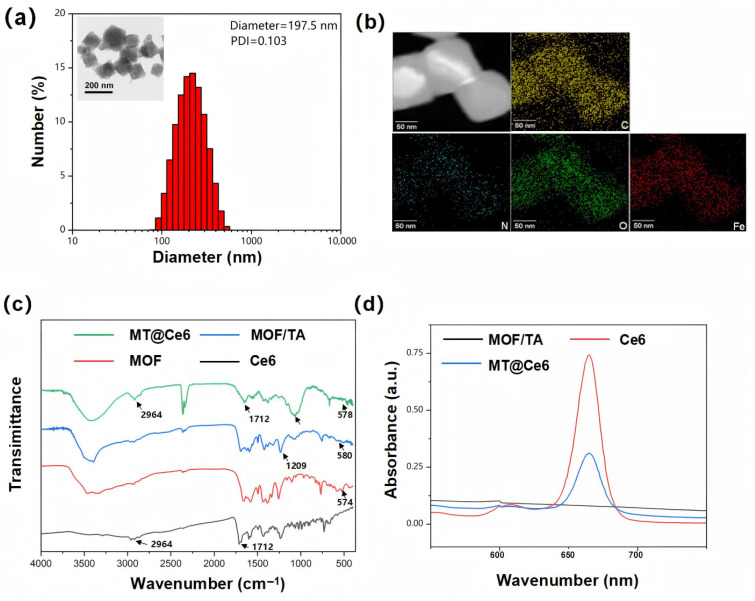
Synthesis and structural validation of MIL-101 (Fe)-NH_2_ and MT@Ce6 nanocomposites. (**a**) TEM image of solvothermally synthesized MOF nanocomposites with corresponding size distribution. (**b**) Dark-field STEM-EDS elemental maps demonstrating spatial uniformity of key constituents. (**c**) Comparative FTIR spectra highlighting characteristic bond vibrations and coordination interactions. (**d**) UV-vis absorption profiles confirming functional component integration.

Building upon prior methodologies, we first adapted an established hydrothermal protocol as the foundation for synthesizing MIL-101 (Fe)-NH_2_ nanocomposites [29,30]. Following systematic optimization of the synthesis parameters, rigorous parametric screening, and subsequent characterization, Protocol No. 4 was ultimately confirmed as the optimal fabrication strategy for MOF synthesis (Table S1). TEM analysis revealed well-dispersed MOF nanocomposites with an average particle size of approximately 197.5 nm (Figure 1a). The results from STEM analysis further confirmed the uniform distribution of carbon, nitrogen, and iron elements within the MOF structure (Figure 1b), validating its successful synthesis.

Next, the MOF/TA nanocomposite was constructed through coordination interactions between the polyphenol groups of TA and Fe^3+^ ions in the MOF. FTIR spectroscopy demonstrated a characteristic Fe-O stretching vibration at 579 cm^−1^ (Figure 1c), consistent with the MOF framework (585 cm^−1^). A significant reduction in the O-H stretching peak intensity at 1209 cm^−1^ (compared to free TA at 1203 cm^−1^) confirmed effective coordination between TA hydroxyl groups and Fe^3+^. Furthermore, in the MT@Ce6 composite the characteristic C–H stretching vibration at 2964 cm^−1^ and the C=O asymmetric stretching band at 1712 cm^−1^ are both preserved, and the UV–vis spectrum (Figure 1d) displays the distinctive Q-band absorption of Ce6 at 660 nm. Taken together, these spectroscopic signatures confirm the successful incorporation of Ce6 into the MOF/TA framework, yielding the MT@Ce6 nanocomposite.

### 3.2. pH-Responsive Degradation and Hydroxyl Radical Generation

**Figure 2:**
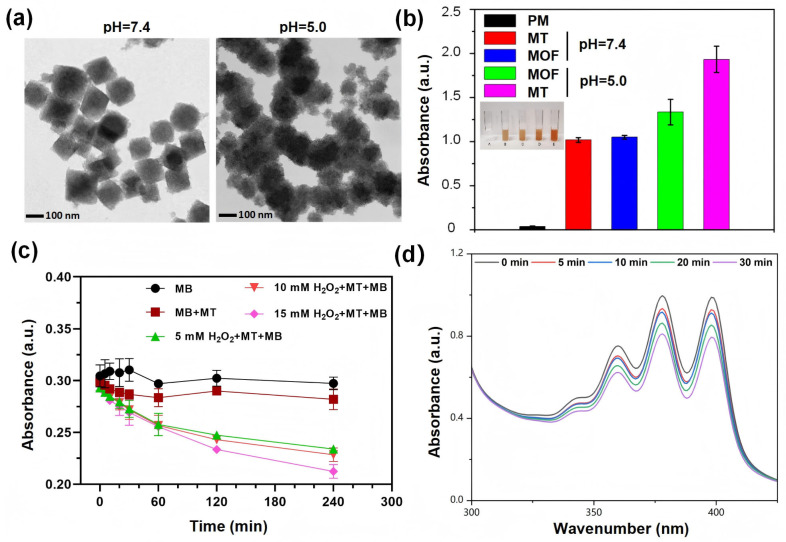
pH-responsive properties and radical generation validation. (**a**) pH-dependent morphological evolution of MOF/TA from cubic to aggregated structures under acidic conditions. (**b**) Phenanthroline colorimetric assays showing enhanced Fe^3+^ release in acidic microenvironments. (**c**) Time-resolved methylene blue decolorization indicating Fenton-driven hydroxyl radical generation. (**d**) Laser-activated ^1^O_2_ production monitored by ABDA absorption attenuation under varied pH conditions. PM, phenanthroline monohydrate; MT, MOF-TA; MOF, MIL-101 (Fe)-NH_2_.

The MOF/TA nanocomposite exhibited pH-dependent structural degradation. Under acidic conditions (pH 5.0), its morphology transitioned from cubic to irregular aggregates, while remaining intact at physiological pH (7.4) for over 12 h (Figure 2a). Phenanthroline (Phen) colorimetric assays revealed enhanced Fe^3+^ release under acidic conditions, as evidenced by the distinct orange-red coloration and stronger absorbance of the MOF/TA + Phen supernatant (Figure 2b). This suggests TA-mediated reduction of Fe^3+^ to Fe^2+^, which exhibits superior Fenton reaction activity. As shown in Figure 2c, MB decolorization was negligible (<5%) in both the MB-only and MB + MOF/TA groups, indicating that MOF/TA alone does not generate •OH. Upon addition of 5, 10, or 15 mM H_2_O_2_, decolorization efficiencies increased to 19.7%, 22.0%, and 28.5%, respectively, demonstrating that MOF/TA catalyzes Fenton chemistry in a H_2_O_2_-concentration-dependent manner. These results support the hypothesis that MOF/TA acts as a pH-responsive nanogenerator for sustained •OH release in acidic microenvironments.

Subsequently, the singlet oxygen generation capability of MT@Ce6 under 660 nm laser irradiation was evaluated in PBS at pH 5.0. ABDA was employed as a selective ^1^O_2_ probe, demonstrating efficient •OH production. A time-dependent decrease in the absorption spectra was observed with prolonged illumination (Figure 2d). Overall, these results provide compelling evidence for the dual functionality of the nanocomposite, featuring pronounced acid sensitivity and enhanced ^1^O_2_ generation efficiency.

### 3.3. ROS Generation and Photodynamic Bactericidal Mechanism

**Figure 3:**
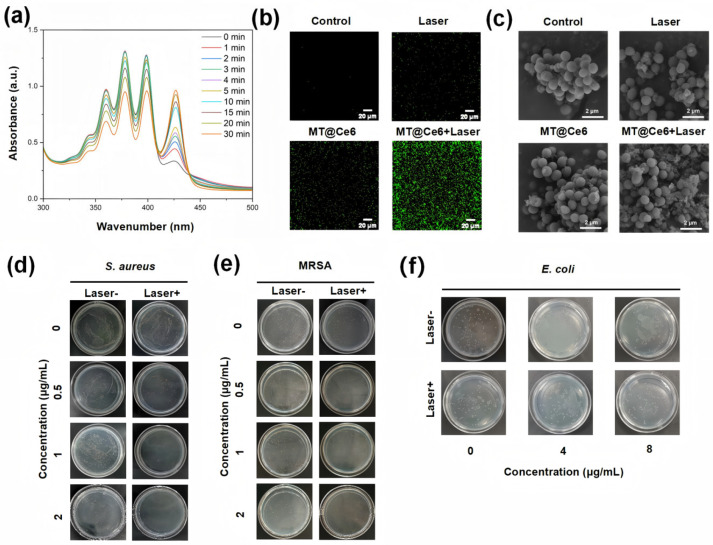
Photodynamic activation and bactericidal mechanism of MT@Ce6. (**a**) Photoactivated ^1^O_2_ generation monitored by time-dependent ABDA absorption attenuation under 660 nm irradiation. (**b**) Fluorescent imaging of intracellular ^1^O_2_ production via DCFH-DA probe. (**c**) FE-SEM micrographs revealing membrane-disruptive effects on *S. aureus* after photodynamic treatment. (**d**–**f**) Photographs of *S. aureus* (**d**), MRSA (**e**), and *E. coli* (**f**) treated with varying concentrations of MT@Ce6.

The bactericidal mechanism of MT@Ce6 was elucidated through multidimensional experiments. Firstly, time-dependent attenuation of ABDA absorption peak at 359 nm confirmed sustained ^1^O_2_ generation by MT@Ce6 under 660 nm irradiation, and the intensity decreased to 70% of the initial value following 30 min of 660 nm irradiation (Figure 3a). The DCFH-DA fluorescence probe further verified the transmembrane action of ROS: no green fluorescence was observed in dark-treated bacteria, while distinct specific fluorescence emerged post 10 min irradiation (Figure 3b). Field-emission scanning electron microscopy (FE-SEM) analysis displayed that the cell morphology phenotypes after irradiation changed obviously, such as severe membrane shrinkage, rupture, and cytoplasmic leakage (Figure 3c), whereas the cells in the control groups maintained intact morphology. This demonstrates that MT@Ce6 converts ground-state oxygen into cytotoxic ^1^O_2_ upon photoactivation, with oxidative effects preferentially disrupting the structural barriers of Gram-positive bacteria, ultimately leading to lytic death. As shown in Figure S1, further validation via APF fluorescence imaging confirmed enhanced ROS generation under irradiation, with the MT@Ce6 + light group exhibiting significantly higher fluorescence intensity compared to other experimental groups. APF is highly responsive to •OH but shows minimal sensitivity to ^1^O_2_ [31], thus serving as a reliable indicator of •OH production. This pronounced ROS signal directly correlated with its superior bactericidal performance, underscoring the critical role of photodynamic activity in the antimicrobial mechanism.

Based on this mechanism, the spread plate method was used to detect its bactericidal efficacy. MT@Ce6 exhibited a concentration-dependent inhibition against *S. aureus* in darkness (0–2 μg/mL), while sterilization rate was significantly enhanced after 660 nm irradiation, attaining a high level of bacterial inhibition at 2 μg/mL (Figure 3d). Notably, this dual-mode effect remained effective against MRSA, and the inactivation efficiency for Gram-positive *S. aureus* was higher than that for Gram-negative *E. coli* (Figure 3e,f). Such selectivity can be attributed to structural differences: the thick peptidoglycan layer of Gram-positive bacteria enhances MOF adhesion and ROS penetration, whereas the outer membrane of Gram-negative bacteria impedes material delivery and attenuates oxidative damage.

### 3.4. In Vivo Antibacterial Efficacy

**Figure 4:**
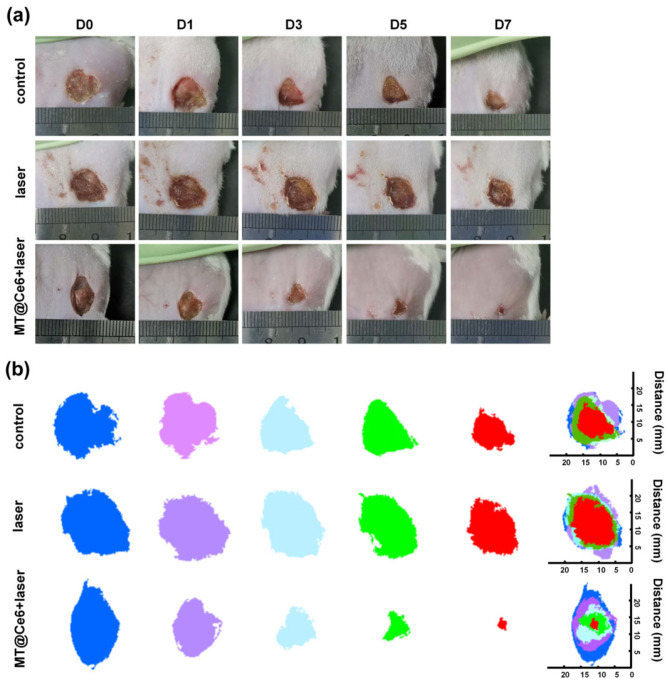
In vivo therapeutic efficacy assessment of MT@Ce6. (**a**) Comparative wound photographs of SA-infected mice across treatment groups at key time points. (**b**) Simulated wound healing progression.

A murine wound infection model was employed to evaluate therapeutic outcomes. As shown in Figure 4a, the control group exhibited delayed healing with persistent erythema and exudation. The laser group showed partial wound area reduction due to light-induced microenvironmental effects. In contrast, the MT@Ce6 + laser group achieved over 95% wound contraction by day 7 (Figure 4b), with complete epithelial regeneration and no signs of secondary infection. Therapeutic outcomes analysis revealed progressive scab formation and near-complete restoration in MT@Ce6 laser-treated wounds within 7 days post-intervention, demonstrating significantly enhanced healing efficacy compared to control group.

## 4. Conclusions

This study demonstrates that the pH-responsive nanophotosensitizer MT@Ce6 significantly enhances antimicrobial PDT through pH-triggered •OH generation. Under acidic conditions, MT@Ce6 shifts ROS dominance from short-lived ^1^O_2_ to highly penetrative •OH, leveraging its superior oxidative capacity for targeted bacterial membrane disruption and biofilm eradication. The nanoplatform integrates intrinsic dark toxicity with light-activated •OH production, achieving >95% inactivation of MRSA at 2 μg/mL under 660 nm irradiation while maintaining pH-dependent selectivity. In vivo evaluations further confirm accelerated wound regeneration (90% contraction within 7 days) through •OH-mediated bacterial clearance, minimizing collateral damage to host tissues.

Benefiting from the acidic microenvironment of infections, this work overcomes critical limitations of conventional photosensitizers, such as limited ROS penetration. By linking material design with recognition of the pathological microenvironment, the pH-responsive •OH amplification strategy establishes a conceptual framework for adaptive antimicrobial platforms. Future research should focus on developing multi-modal systems that respond to complementary biomarkers (e.g., specific enzymes or hypoxic conditions), thereby advancing precision therapies for polymicrobial and chronic infections.

## Data Availability

Data are contained within the article and Supplementary Materials.

## Supplementary Materials

The following supporting information can be downloaded at: https://www.mdpi.com/article/10.3390/nano15141075/s1, Table S1: Different synthesis parameters and resultant particle metrics in MOF fabrication; Figure S1: Comparative analysis of ^1^O_2_ levels across bacterial treatment groups via APF fluorescence imaging.

## Abbreviations

The following abbreviations are used in this manuscript:

TA	tannic acid
PDT	photodynamic therapy
MOF	metal–organic frameworks
MRSA	methicillin-resistant *Staphylococcus aureus*
ROS	reactive oxygen species
