# Supplementary material for: pH-Responsive Nanophotosensitizer Boosting Antibacterial Photodynamic Therapy by Hydroxyl Radical Generation
Source: Nanomaterials (Basel). 2025 Jul 10;15(14):1075. doi: 10.3390/nano15141075 (PMC12298318; doi:10.3390/nano15141075)
Supplement: Supplementary file 1 [file nanomaterials-15-01075-s001.zip › nanomaterials-3716648-supplementary.pdf]

# Supplementary Materials

**Table S1.** Different synthesis parameters and resultant particle metrics in MOF fabrication.

| Group | Reactant Quantity | DMF Volume (mL) | Reaction Conditions (Temperature/Time) | Mean Particle Size (nm) | PDI |
|---|---|---|---|---|---|
| 1 | 1.8912 g $FeCl_3 \bullet 6H_2O$<br>0.6339 g BDC-$NH_2$ | 43 mL | 180 ℃<br>24 h | 1511 nm | 0.8087 |
| 2 | 0.9456 g $FeCl_3 \bullet 6H_2O$<br>0.3169 g BDC-$NH_2$ | 21.5 mL | 180 ℃<br>24 h | 1072 nm | 0.38 |
| 3 | 0.45 g $FeCl_3 \bullet 6H_2O$<br>0.6 g BDC-$NH_2$ | 15 mL | 110 ℃<br>24 h | 315 nm | 0.123 |
| 4 | 0.45 g $FeCl_3 \bullet 6H_2O$<br>0.6 g BDC-$NH_2$ | 15 mL | 110 ℃<br>12 h | 197.5 nm | 0.103 |

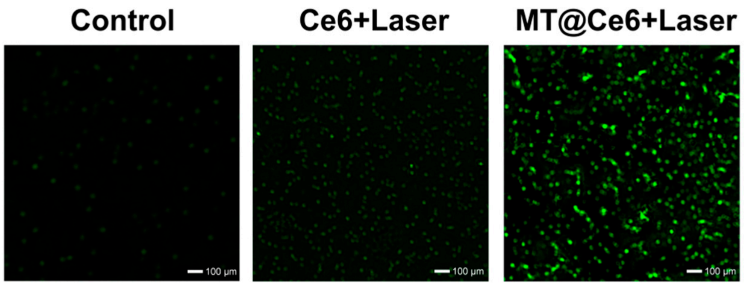


**Figure S1.** Comparative analysis of $^1O_2$ levels across bacterial treatment groups via APF fluorescence imaging.
